# The efficacy and safety of Endostar combined with chemoradiotherapy for patients with advanced, locally recurrent nasopharyngeal carcinoma

**DOI:** 10.18632/oncotarget.5271

**Published:** 2015-09-15

**Authors:** Ying Guan, Anchuan Li, Weiwei Xiao, Shuai Liu, Binbin Chen, Taixiang Lu, Chong Zhao, Fei Han

**Affiliations:** ^1^ Department of Radiotherapy Oncology, Sun Yat-Sen University Cancer Center, State Key Laboratory of Oncology in South China, Collaborative Innovation Center for Cancer Medicine, Guangzhou 510060, P. R. China; ^2^ Department of Radiation Oncology, Affiliated Cancer Hospital of Guangxi Medical University, Cancer Institute of Guangxi Zhuang Autonomous Region, Nanning, Guangxi 530021, P.R. China; ^3^ Department of Radiation Oncology, Fujian Medical University Union Hospital, Fuzhou 350009, P. R. China; ^4^ Department of Radiotherapy Oncology, The Sixth Affiliated Hospital of Sun Yat-Sen University, Guangzhou 510655, P. R. China

**Keywords:** Endostar, nasopharyngeal carcinoma, recurrence, chemotherapy, intensity-modulated radiotherapy

## Abstract

**Purpose:**

To evaluate the short-term efficacy and safety of recombinant human endostatin (Endostar) combined with chemoradiotherapy for the treatment of advanced, locally recurrent nasopharyngeal carcinoma (NPC).

**Materials and Methods:**

Between March 2010 and October 2013, a total of 22 patients with stage rIII-IVb locally recurrent NPC underwent salvage radiotherapy with Endostar in Sun Yat-Sen University Cancer Center. Intensity-modulated radiotherapy (IMRT) was delivered. Platinum-based chemotherapy was used in a neoadjuvant protocol. Endostar was continuously administered intravenously (105 mg/m^2^) for 14 days (Days 1–14) from the first day of treatment during a 21-day cycle. Tumor response and treatment toxicities were observed.

**Results:**

Until January 2014, the median follow-up time was 13 months (range, 4–41 months). All patients completed the planned radiotherapy. A complete response was achieved in 20 patients, and a partial response was achieved in 2 patients. The incidence of grade 3–5 late radiation injury in this study was 50% (11/22) and that of nasopharyngeal mucosal necrosis was 31.8% (7/22).

**Conclusions:**

Endostar combined with chemoradiotherapy may be effective in decreasing both the incidence of nasopharyngeal mucosal necrosis. Studies with a larger sample size and longer follow-up are warranted.

## INTRODUCTION

Recurrent nasopharyngeal carcinoma (NPC) has a unique set of pathological and clinical characteristics. The management of recurrent NPC remains a challenging clinical problem. There is no standardized protocol or guideline for the management of locally recurrent NPC. The major salvage therapies of local recurrent NPC include local treatment and systemic chemotherapy. Aggressive local treatment methods, including surgery, stereotactic radiotherapy and brachytherapy, are effective in highly selected patients with small recurrent lesions within the nasopharynx, whereas chemotherapy alone achieves only a palliative effect for patients with a bulky tumor burden or for those with poor physical performance. All of the above-mentioned treatments are usually limited by the size, extent, and location of the tumor, as well as by patient intention and consultation of radiation oncologists and surgeons. In general, radiotherapy (RT) and/or chemotherapy are suitable for the treatment of all recurrent T classification cases, but salvage surgery is performed for early recurrent disease (T1-T2a) or for certain highly selected recurrent T2b cases confined to the superficial parapharyngeal space and for recurrent T3 disease confined to the base wall of the sphenoid sinus [1, 2]. Therefore, external beam radiotherapy is still an important modality and is sometimes the sole choice for the salvage treatment of locally recurrent NPC patients.

Intensity-modulated radiotherapy (IMRT) has gradually been accepted as the preferred choice for the re-irradiation of locally recurrent NPC because of its dosimetric superiority. However, even with IMRT, re-irradiation is associated with a high incidence of late complications and, consequently, toxicity-related death. In recent years, the 5-year local control rate of the patients with locally recurrent NPC who were treated with IMRT was enhanced to 80.7%-85.8%, but the 5-year OS rate was only 38%-44.9% [1, 3, 4].

After the initial course of treatment, the sensitivity of locally recurrent tumor tissue to chemoradiotherapy would decrease significantly due to tissue fibrosis and vascular changes. To improve outcome, a minimal re-irradiation dose combined with effective agents is another choice. In recent years, targeted therapy has become a promising anti-tumor treatment, ranked after the current three primary anti-tumor methods: surgery, radiotherapy and chemotherapy. When combined with surgery, radiotherapy or chemotherapy, targeted therapy has shown additive and synergistic effects [5–9].

Endostar is a new recombinant human endostatin injection synthesized in China and is a multi-target tumor cell inhibitor. Endostar can inhibit tumor growth primarily through direct inhibition of vascular endothelial cell proliferation via multiple targets such as vascular endothelial growth factor (VEGF), vascular endothelial growth factor receptor-2 (VEGFR-2) and platelet-derived growth factor receptor (PDGFR). It can also exert an antitumor effect via vascular normalization [10]. In the clinical setting, Endostar has already shown encouraging results for some tumors [11, 12].

On the basis of apparent benefits of Endostar in other malignancies, we implemented the use of Endostar with advanced locally recurrent NPC patients treated at our department. We conducted this retrospective study to report the outcome in these patients.

## RESULTS

### Efficacy

The baseline characteristics and treatment outcomes of the 22 patients are listed in Table 1. Until January 2014, the median follow-up period was 13 months (range, 4–41 months). All patients completed the planned radiotherapy. At the completion of IMRT, complete responses (CR) were achieved in 20 patients and partial responses (PR) in 2 patients. Residual nasopharyngeal tumor in one patient was noted to have continuously shrunk in subsequent follow-up, and the patient underwent observation. Residual cervical lymph nodes in the other patient did not continuously shrink over time; therefore, the patient underwent surgical excision and postoperative radiotherapy 4 months after the completion of IMRT.

**Table 1:** Demographic and baseline characteristics of patients

Case	Sex	Age	Recurrence interval	rT stag	rN stage	Histology	Neoadjuvant chemotherapy	Concurret E	IMRT prescribed dose	GTV nx	Maximal dose of GTV nx	Mean dose of GTV nx	Fractionation of GTVnx	Necrosis of nasopharyngeal mucosa	R/DM	Cause of death
		(years)	(months)					(Cycles)	(Gy/F)	(cm^3^)	(Gy)	(Gy)	(Gy)	(Yes/No)		
1	M	55	120	rT3	rN0	-	TP × 2+E × 2	E × 2	60/32	75.00	65.07	61.93	1.94	No	/	/
2	M	43	51	rT3	rN0	III	TP × 2+E × 2	E × 2	62/32	9.40	65.18	63.98	2.00	No	/	/
3	M	44	51	rT3	rN3b	III	TP × 2+E × 2	E × 2	62/32	25.80	69.27	65.67	2.05	No	/	/
4	M	56	18	rT3	rN1b	III	TP × 5+E × 4	E × 2	64/32	28.40	69.13	66.24	2.07	No	/	REP
5	M	61	10	rT3	rN0	III	PF × 2+E × 2	E × 2	64/32	15.60	71.32	67.07	2.10	No	/	/
6	M	56	80	rT4	rN0	III	TP × 2+E × 2	E × 2	64/32	40.04	71.12	67.17	2.10	No	/	/
7	M	43	36	rT4	rN1b	III	TP × 3+E × 3	E × 2	65/32	38.98	68.71	64.59	2.02	No	DM	DM
8	M	44	42	rT3	rN0	III	TP × 2+E × 2	E × 3	66/33	20.71	73.00	69.68	2.11	No	/	/
9	M	50	44	rT3	rN0	III	TP × 2+E × 2	E × 2	66/32	18.60	71.91	69.21	2.16	Yes	/	Hemorrhage
10	F	39	12	rT4	rN0	III	TP × 2+E × 2	E × 2	66/30	19.91	66.69	63.61	2.12	Yes	/	/
11	M	43	44	rT4	rN0	-	TP × 2+E × 2	E × 3	68/32	40.27	72.53	67.67	2.11	No	/	/
12	F	39	29	rT4	rN3b	-	TPF × 5	E × 2	64/29	46.10	71.09	57.33	1.98	No	/	/
13	M	36	18	rT4	rN0	III	TPF × 2	E × 2	64/29	61.70	70.58	66.33	2.29	Yes	/	/
14	F	49	48	rT4	rN0	III	TPF × 3	E × 2	64/28	62.70	69.15	65.84	2.35	No	/	/
15	M	28	31	rT3	rN1b	III	GP × 3+PF × 3	E × 2	64/30	47.20	71.08	64.15	2.14	No	/	/
16	F	29	39	rT3	rN3b	III	TP × 2+PF × 3+TPF × 3	E × 1	66/30	150.60	75.28	69.75	2.33	Yes	/	/
17	M	39	9	rT4	rN1b	-	GP × 6	E × 2	66/30	65.60	72.40	69.22	2.31	Yes	R+ DM	R+ DM
18	M	55	18	rT3	rN0	III	TP × 2+E × 1	/	62/32	29.50	66.12	54.23	1.69	No	/	/
19	F	48	13	rT4	rN0	-	/	E × 2	64/29	41.60	70.81	67.14	2.32	Yes	/	/
20	M	46	69	rT3	rN0	II	/	E × 2	64/32	16.30	68.41	65.10	2.03	No	/	/
21	M	48	19	rT3	rN0	III	/	E × 2	65/29	28.70	71.73	68.89	2.38	Yes	/	/
22	M	46	9	rT3	rN0	III	/	E × 2	66/29	28.00	71.93	70.04	2.42	No	/	/

Abbreviations: WHO, World Health Organization; M, Male; F, Female; TP, Docetaxel + Cisplatin; Gy, Gray; F, Fractionation; GP, Gemcitabine + Cisplatin; PF, Cisplatin + 5-Flurouracil; TPF, Docetaxel + Cisplatin + 5-Flurouracil; E, Endostar, Recombinant human endostatin injection; IC, Induction chemotherapy; IMRT, Intensity-modulated radiotherapy; GTVnx, Gross tumor volume of nasopharynx; REP, Radiation Encephalopathy; R, recurrence; DM, Distance Metastasis.

Treatment failures were as follows: regional lymph node recurrence and multi-site metastasis including the liver, lung and mediastinum in 1 patient; and liver metastasis in 1 patient. A total 4 patients died. Two deaths were attributed to tumor metastasis, 1 to nasopharyngeal hemorrhage and 1 to radiation temporal lobe necrosis. The 1-year and 2-year OS, local-regional failure free survival (LRFFS), distant metastasis-free survival (DMFS) and progression-free survival (PFS) rates were as follows: OS, 93.3% and 66.4%, respectively; LRFFS, 89.3% and 78.1%, respectively; DMFS, 90.0% and 78.8%, respectively; and PFS, 92.3% and 52.7%, respectively.

### Toxicity

No grade 5 toxicity (death) occurred during treatment. Acute ≥ grade 3 toxicities included cardiotoxicity (1 patient), mucositis (3 patients) and myelosuppression (3 patients). Late ≥ grade 3 toxicities were documented as follows: radiation encephalopathy in 4 patients; and nasopharyngeal mucosal necrosis in 7 patients (among these 1 patient experienced nasopharyngeal hemorrhage). The clinical characteristics of the patients with or without necrosis of the nasopharyngeal mucosa are listed in Table 2. The median fractionation dose of patients with nasopharyngeal mucosal necrosis was significantly higher than that of patients without necrosis (2.20 Gy versus 2.00 Gy, *p* = 0.01).

**Table 2:** Clinical characteristics of patients with or without nasopharyngeal mucosa necrosis

Characteristics	Necrosis of nasopharyngeal mucosa		χ^2^/*t*	*p*
	Yes (*n* = 7)	No (*n* = 15)		
Sex			2.37	0.12
Male	4	13		
Female	3	2		
Age(years) *			1.58	0.13
Median	39	46		
Range	29–50	28–61		
Recurrence interval (Months)*			1.85	0.08
Median	18	42		
Range	9–44	9–120		
rT stage			1.12	0.29
rT3	3	10		
rT4	4	5		
rN stage			0.11	0.95
rN0	5	10		
rN1b	1	3		
rN3b	1	2		
GTVnx (cm^3^)*		−	1.51	0.15
Median	41.60	29.50		
Range	18.60–150.60	9.40–75.00		
Mean ± SD	55.24 ± 46.05	34.93 ± 17.93		
Maximal dose of GTVnx (Gy)*		−	1.55	0.14
Median	71.73	69.27		
Range	66.69–75.28	65.07–73.00		
Mean ± SD	71.34 ± 2.57	69.54 ± 2.54		
Mean dose of GTVnx (Gy)*			−1.76	0.09
Median	68.89	65.67		
Range	63.61–69.75	54.23–70.04		
Mean ± SD	67.74 ± 2.20	64.71 ± 4.23		
Fractionation of GTVnx (Gy)*			−2.70	**0.01**
Median	2.20	2.00		
Range	2.06–2.24	1.88–2.29		
Mean ± SD	2.19 ± 0.06	2.05 ± 0.13		
Courses of Endostar(Cycles)*			1.81	0.09
Median	2	4		
Range	1–4	2–6		

Abbreviations: GTVnx, Gross tumor volume of nasopharynx; Gy, Gray; SD, Standard Deviation

* *t* test was used for comparison.

## DISCUSSION

Salvage treatment of locally recurrent NPC is a very difficult issue. Patients with early stage disease may be curable. In contrast, the treatment options for advanced locally recurrent NPC patients (rT3-T4) are very limited, and re-irradiation is the only choice in most circumstances. Poon et al [13] adopted concomitant chemoradiotherapy with adjuvant chemotherapy to treat 35 locally recurrent NPC patients, 66% of whom were staged as rT3-T4, and the 2-year OS and PFS rates were only 45% and 23%, respectively. Chua et al [14]. reported that 1-year locoreginal progression-free, distant metastasis-free and OS rates of 56%, 90% and 63% respectively, after a median follow-up time of 11 months; the 1-year local progression-free rate of rT4 in NPC patients was only 35% versus 100% for rT1–3 patients. In 2004, we began to treat locally recurrent NPC patients using IMRT, and we previously reported that the 2-year OS rate of rT1–2 patients was 85%, whereas that of rT3–4 patients was only 56% [1, 3, 15]. Taken together, the treatment of advanced locally recurrent NPC is a significant challenge for oncologists.

In recent years, targeted therapy has become another promising treatment method for malignancies. Epidermal growth factor receptor (EGFR) and VEGFR are strongly expressed in head and neck squamous cell carcinomas (HNSCC), including NPC. Several studies of HNSCC have demonstrated that a variety of targeted drugs perform anti-tumor activity and yield encouraging clinical outcomes by blocking these specific receptors. Chan et al [16] conducted a phase II study to evaluate the activity and safety of cetuximab plus carboplatin in 60 patients with advanced locally recurrent or metastatic NPC who experienced treatment failure after platinum-based chemotherapy. The results showed an overall response rate (RR) and disease control rate (DCR) of 11.7% and 60.0%, respectively. Cohenet al [17] performed a phase I/II study to treat 48 recurrent or metastatic HNSCC patients using Erlotinib and Bevacizumab. The objective RR and DCR were 15% and 46%, respectively. Argiris et al [18] reported the treatment outcomes of a phase II study to evaluate the efficacy of pemetrexed and Bevacizumab in 40 recurrent or metastatic HNSCC patients. The objective RR and DCR were 30% and 86%, respectively.

Endostatin is a broad-spectrum angiogenesis inhibitor and tumor suppressor. Ye et al [19] performed a randomized, open-label, phase II, multicenter clinical trial in patients with advanced HNSCC and recurrent or metastatic NPC using paclitaxel and cisplatin with or without adenovirus-encapsulated endostatin (E10A) administered by intratumoral injection. E10A plus chemotherapy were well tolerated. The authors detected an objective RR that rose to 39.7% in the E10A group, comparing favorably with that reported in trials using Bevacizumab [17, 18]. The authors also assessed molecular biomarkers related to angiogenesis in patient serum before and after the first administration of E10A. The serum VEGF levels in E10A treated patients were significantly lower in the CR+PR groups than in the SD (stable disease) +PD (progressive disease) groups, which supports the hypothesis that endostatin suppresses VEGF-stimulated endothelial cell proliferation, migration, and tumor angiogenesis.

Endostar (recombinant human endostatin injection), a type of human angiogenesis inhibitor, exerts anti-tumor effects by inhibiting the function of endothelial cells and reducing tumor angiogenesis [10]. To our knowledge, no data have been previously reported regarding the efficacy and safety of Endostar for the treatment of recurrent NPC. Our retrospective analysis showed that Endostar plus IMRT yielded quite promising tumor control and survival results for advanced locally recurrent NPC patients. The RR, CR, 1- and 2-year OS rates reached 100.0%, 90.9%, 93.3% and 66.4%, respectively, which were significantly superior to results reported in the literature [1, 3, 13, 20]. These findings exceeded our expectations and suggested the potential efficacy of Endostar in the treatment of advanced locally recurrent NPC patients.

In recent years, the 5-year local control rate of the patients with locally recurrent NPC treated with IMRT was enhanced to 80.7%-85.8%, but the 5-year OS rate was only 38%-44.9% [1, 3, 4]. Late radiation injury is the major reason for treatment failure and has become the most important concern in the re-irradiation of locally recurrent NPC patients. Han et al [1] reported that the incidence of grade 3–5 radiation injury was 70.7% after the second course of radiotherapy in recurrent NPC (rT3–4, 75.3%), of which 40.6% suffered from nasopharyngeal mucosal infection and/or necrosis (more than 70% in rT3 and rT4 patients). Among these patients, severe cases would develop deadly nasopharyngeal hemorrhage. In addition, Qiu et al [20] reported that 70 patients with radiologic or pathologically proven locally recurrent NPC were treated with IMRT. Only fifty-seven percent were staged as rT3–4. Sixty-five patients received the planned radiation therapy (median dose, 70 Gy), and 5 patients had their treatment terminated between 50 and 60 Gy because of acute mucositis. Late grade 3–4 toxicities were noted in 25 patients (35.7%). Eleven patients (15.7%) had posterior nasal space ulceration, 17 (24.3%) experienced cranial nerve palsies, 12 (17.1%) had trismus, and 12 (17.1%) experienced deafness.

**Figure 1:**
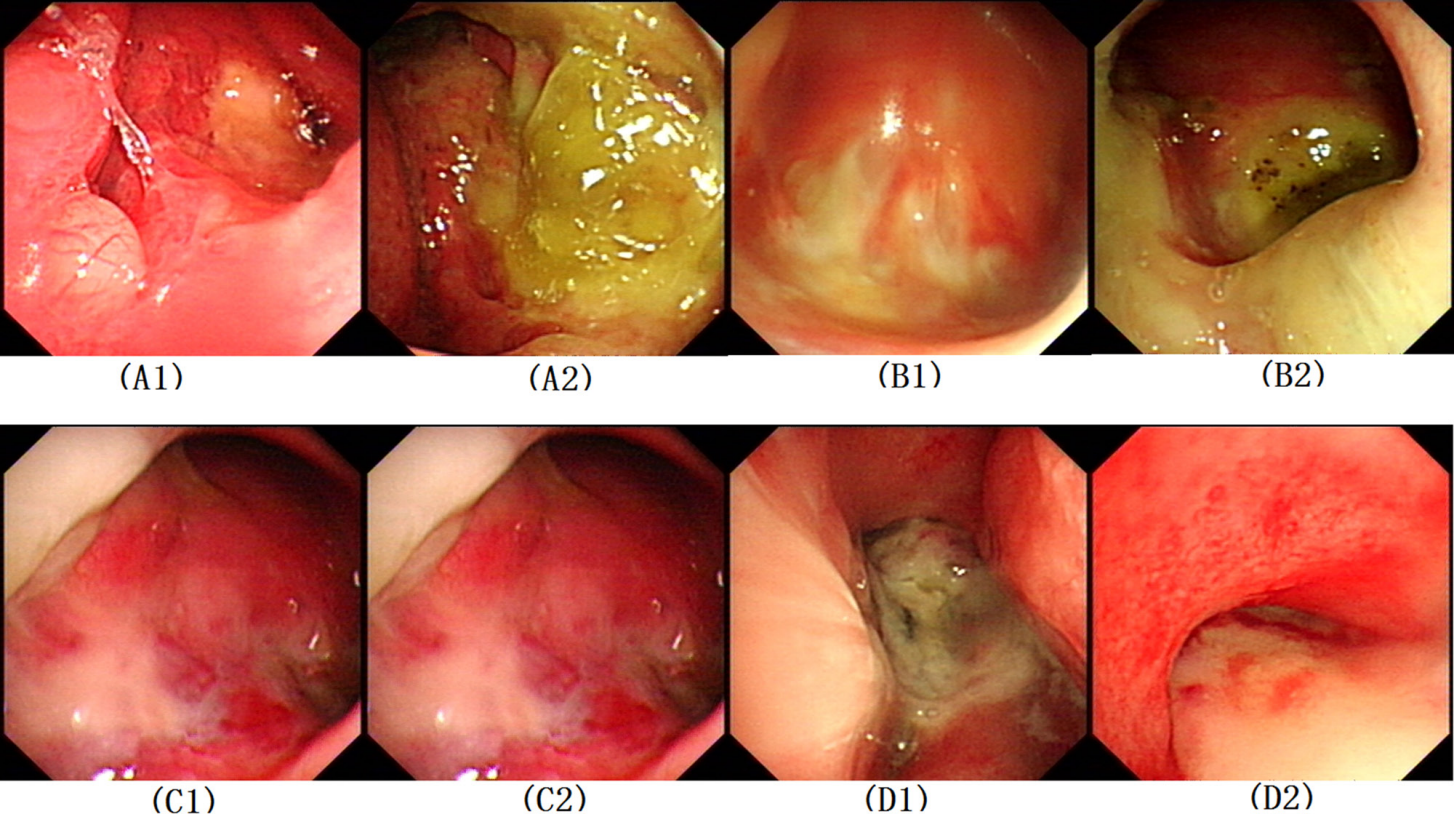
Nasopharyngeal images of recurrent nasopharyngeal carcinoma patients Patients with IMRT alone: **A1.** Before re-irradiation of patient A, **A2.** End of re-irradiation of patient A, **B1.** End of re-irradiation of patient B, **B2.** 2 years after re-irradiation of patient B; Patients with Endostar^®^ + IMRT, **C1.** End of re-irradiation of patient C, **C2.** 1 month after re-irradiation of patient C, **D1.** End of re-irradiation of patient D, **D2.** 18 months after re-irradiation of patient D. IMRT, Intensity-modulated radiotherapy.

The incidence of late grade 3–5 radiation injury in this study was 50% (11/22), and the incidence of nasopharyngeal mucosal infection and/or necrosis was 31.8% (7/22), which is substantially lower than that noted in historic data regarding advanced recurrent NPC. Endoscopic examination results of the patients previously treated with IMRT alone in our center and treated with Endostar + IMRT in this study are shown in Figure 1.

Endothelial cell apoptosis induced by radiotherapy can damage the integrity of the vascular barrier, resulting in tissue edema and hypoxia. Meanwhile, hypoxia also leads to VEGF upregulation; consequently, this situation contributes to increased vascular permeability and tissue necrosis [21–23]. Endostar can participate in all tumor angiogenic processes. Endostar combined with chemoradiotherapy may be effective in decreasing both the incidence and degree of nasopharyngeal mucosal necrosis. First, Endostar could normalize abnormal vessels surrounding the tumor in the irradiation field and improve the blood supply to necrotic tissue. Second, Endostar could decrease the immature vascular number to reduce oxygen consumption, enhance hypoxic tolerance, reduce inflammatory exudation and strengthen the recovery capability of necrotic tissue by downregulation of VEGF activity [10, 11]. Zheng et al [25] determined that Endostar could increase radiotherapy sensitivity and attenuate radiation-induced lung injury (RILI) by vascular normalization and downregulation of the expression of the inflammatory mediator-transforming growth factor β1 (TGFβ1). Chen et al [26] found that Endostar could decrease liver inflammation and hepatocyte necrosis in the CCl_4_-induced liver fibrosis model. Their data indicate that Endostar might play a role in counteracting liver fibrosis and hepatocyte necrosis through the TGFβ and VEGF transduction pathways.

The time course of “vascular normalization” is also a crucial factor that should be taken into account when Endostar and chemoradiotherapy are combined. Peng et al [11] demonstrated that the Endostar vascular normalization time window was within approximately 3 days after Endostar administration in the CNE-2 and 5–8F NPC xenograft models. In addition, the combination of radiation with Endostar showed enhanced anti-tumor effects during the normalization time induced by delivering Endostar. The authors also investigated the underlying mechanisms that suggested that during the normalization window, Endostar might increase the pericyte coverage of NPC tumor vessels via upregulation of a powerful endogenous anti-angiogenic and antitumor factor-Pigment epithelium-derived factor (PEDF) and downregulation of VEGF, thus inhibiting VEGF signaling that can be attributed to the local increase in oxygenation and decrease in tissue necrosis and edema.

In addition, the median fractionation dose of the patients developing nasopharyngeal mucosal necrosis (2.20 Gy) was significantly higher than the dose of those without necrosis (2.00 Gy), implying that a high fractionation dose may be one of the important and notable causes of nasopharyngeal mucosal necrosis.

For advanced locally recurrent NPC, Endostar combined with chemoradiotherapy yielded better short-term efficacy and good tolerance. Endostar may alleviate nasopharyngeal mucosal necrosis after the second course of radiotherapy.

A prospective study is warranted to confirm the results we obtained from our retrospective analysis and we have already begin to work on it.

## MATERIALS AND METHODS

### Patient selection

The inclusion criteria were as follows: (1) locally recurrent III-IVb NPC, according to the 2009 7^th^ edition of American Joint Cancer Committee on Cancer staging system; (2) no evidence of distant metastases at diagnosis; (3) at least 6 months after the end of initial course of radiotherapy; (4) Karnofsky performance status score ≥ 70; (5) leukocytes ≥ 4.0 × 10^9^/L, neutrophils ≥ 2.0 × 10^9^/L, platelets ≥ 100 × 10^9^/L and hemoglobin ≥ 100 g/L; (6) at least 18 years old with normal hepatic and renal function; (7) patients who have received Endostar combined with radiotherapy/chemotherapy; and (8) provided written informed consent.

Among all the locally recurrent NPC patients who received treatment from March 2010 to October 2013 in our Center, a total of 22 patients matched the criteria. There were 5 females and 17 males, with a median age of 45 years (range, 28–61 years). Magnetic resonance imaging (MRI) of nasopharynx and neck was performed for the staging evaluations. Positron emission tomography computed tomography (PET-CT) was performed at the physician's discretion. Restaging demonstrated rIII, rIVa, and rIVb in 11, 8, and 3 patients, respectively.

### Treatment

All patients underwent IMRT. An MRI with or without PET-CT showed that gross tumor volume (GTV) was contoured. The clinical target volume (CTV) was delineated, including the GTV plus a 1–1.5-cm margin. The CTV also included the entire nasopharynx and the positive lymph node regions. When the GTV was adjacent to critical organs at risk (OARs), for example, the spinal cord, temporal lobe or brainstem, the margin of the CTV was adjusted to no more than 3 mm, depending on the proximity of critical structures. A detailed description of OARs contouring and the delivery procedure of IMRT are provided in our previous reports [1]. The median prescribed dose of IMRT was 64 Gy (60–68 Gy)/32 fractions (28–33 fractions), and the median fractionated prescribed dose was 2.10 Gy (1.88–2.29 Gy).

Among the 22 patients, 18 received cisplatin-based neoadjuvant chemotherapy. Details of the chemotherapy regimens are shown in Table 1.

Endostar was continuously administered intravenously at a dose of 105 mg/m^2^ for 14 days (Days 1–14) from the first day of treatment during a 21-day cycle. The detailed schedules of Endostar are shown in Table 1.

### Response and toxicity assessment

Short-term efficacy was assessed 3 months after the completion of IMRT. The residual tumor was evaluated according to clinical examinations (including nasopharyngeal endoscopy) and MRI. Response Evaluation Criteria in Solid Tumors version 1.1 (RECIST v1.1) was used to assess the treatment response of recurrent soft tumor lesions within the nasopharyngeal cavity.

Acute toxicities were evaluated according to the Common Toxicity Criteria 3.0 of the American National Cancer Institute; late toxicities were graded according to the Radiation Therapy Oncology Group Late Radiation Morbidity Scoring Scheme. Evaluation of acute toxicity (occurring between the start of treatment and 90 days after the end of radiotherapy) was performed once a week and late toxicity (occurring > 90 days after the end of radiotherapy) was conducted at least once every 3 months.

### Follow up

The first follow-up visit was evaluated after IMRT completion. Afterwards, the patients were followed up at 1 and 3 months post-completion, and then every 3 months for the first 3 years and every 6 months thereafter.

OS was defined as the time from the first day of treatment to the date of death or the final follow-up. LRFFS was defined as the time from the first day of treatment until the date of local or regional failure. DMFS was defined as the time from the first day of treatment until the date of distant metastasis. PFS was defined as the time from the first day of treatment until the date of local/regional recurrence or distant metastasis or death.

### Statistical analyses

SPSS 21.0 statistical package was used to analyze the data. The OS, LRFFS, DMFS and PFS rates were analyzed using the Kaplan-Meier method. Log-rank tests were used to detect differences between the groups. The differences between continuous variables were compared with *t* test. The differences between categorical variables were compared with the Chi-squared test. All statistical tests were two-sided, and *p* < 0.05 was considered significant.
